# The role of vascular dementia associated genes in patients with Alzheimer's disease: A large case–control study in the Chinese population

**DOI:** 10.1111/cns.13730

**Published:** 2021-09-22

**Authors:** Xuewen Xiao, Lina Guo, Xinxin Liao, Yafang Zhou, Weiwei Zhang, Lu Zhou, Xin Wang, Xixi Liu, Hui Liu, Tianyan Xu, Yuan Zhu, Qijie Yang, Xiaoli Hao, Yingzi Liu, Junling Wang, Jinchen Li, Bin Jiao, Lu Shen

**Affiliations:** ^1^ Department of Neurology Xiangya Hospital Central South University Changsha China; ^2^ National Clinical Research Center for Geriatric Disorders Central South University Changsha China; ^3^ Department of Geriatrics Xiangya Hospital Central South University Changsha China; ^4^ Engineering Research Center of Hunan Province in Cognitive Impairment Disorders Central South University Changsha China; ^5^ Hunan International Scientific and Technological Cooperation Base of Neurodegenerative and Neurogenetic Diseases Changsha China; ^6^ Key Laboratory of Hunan Province in Neurodegenerative Disorders Central South University Changsha China; ^7^ Department of Radiology Xiangya Hospital Central South University Changsha China; ^8^ Key Laboratory of Organ Injury, Aging and Regenerative Medicine of Hunan Province Changsha China

**Keywords:** Alzheimer's disease, Chinese population, genes, vascular dementia

## Abstract

**Aim:**

The role of vascular dementia (VaD)‐associated genes in Alzheimer's disease (AD) remains elusive despite similar clinical and pathological features. We aimed to explore the relationship between these genes and AD in the Chinese population.

**Methods:**

Eight VaD‐associated genes were screened by a targeted sequencing panel in a sample of 3604 individuals comprising 1192 AD patients and 2412 cognitively normal controls. Variants were categorized into common variants and rare variants according to minor allele frequency (MAF). Common variant (MAF ≥ 0.01)‐based association analysis was conducted by PLINK 1.9. Rare variant (MAF < 0.01) association study and gene‐based aggregation testing of rare variants were performed by PLINK 1.9 and Sequence Kernel Association Test‐Optimal (SKAT‐O test), respectively. Age at onset (AAO) and Mini‐Mental State Examination (MMSE) association studies were performed with PLINK 1.9. Analyses were adjusted for age, gender, and *APOE* ε4 status.

**Results:**

Four common *COL4A1* variants, including rs874203, rs874204, rs16975492, and rs1373744, exhibited suggestive associations with AD. Five rare variants, *NOTCH3* rs201436750, *COL4A1* rs747972545, *COL4A1* rs201481886, *CST3* rs765692764, and *CST3* rs140837441, showed nominal association with AD risk. Gene‐based aggregation testing revealed that *HTRA1* was nominally associated with AD. In the AAO and MMSE association studies, variants in *GSN*, *ITM2B*, and *COL4A1* reached suggestive significance.

**Conclusion:**

Common variants in *COL4A1* and rare variants in *HTRA1*, *NOTCH3*, *COL4A1*, and *CST3* may be implicated in AD pathogenesis. Besides, *GSN*, *ITM2B*, and *COL4A1* are probably involved in the development of AD endophenotypes.

## Graphical abstract

In this study, we systematically explored the relationship between Alzheimer's disease (AD) and vascular dementia (VaD)‐associated genes in a total of 3604 individuals in mainland China. The common variant association test revealed that common variants in *COLA41* were nominally associated with AD. Gene‐based association analysis indicated that *HTRA1* may contribute to the etiology of AD. The rare variant association test showed that variants in *NOTCH3*, *COL4A1*, and *CST3* exhibited nominal association with AD risk. Additionally, variants in *GSN*, *ITM2B*, and *COL4A1* may be involved in the development of AD endophenotypes.

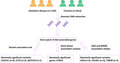

## 1 INTRODUCTION

Dementia is characterized by progressive cognitive impairment and ultimately impaired independent living. The number of dementia cases was estimated at 50 million in 2018 and is expected to triple by 2050. In China, about 15 million individuals aged 60 years or older have dementia, imposing a huge burden on society and family.^1^ Alzheimer's disease (AD) is the most prevalent dementia type worldwide, accounting for approximately 60%–80% of all dementia cases.^2^ The etiology and pathology of AD are complex and remain elusive. In addition to AD, vascular dementia (VaD) is another major subtype of dementia and accounts for 15%–20% of dementia patients in Western countries. VaD refers to dementia caused mainly by vascular pathology. Typically, patients with VaD present with memory problems and executive dysfunction.^3^ It is estimated that around 30% of dementia cases are diagnosed as VaD in Asia.^4^

Vascular pathology coexists in a large proportion of AD cases and reduces the threshold for dementia.^5^ Emerging evidence implicates cerebral microbleeds, white matter lesions, increased blood–brain barrier permeability, attenuated cerebral blood flow, and diminished neurovascular coupling in the development of AD.^6^, ^7^, ^8^ Microvascular alterations, such as increased capillary tortuosity and capillary rarefaction, also exist in AD brains.^9^ Additionally, epidemiological studies suggested that AD and VaD share similar risk factors, including hypertension, obesity, and diabetes.^10^ Vascular risk factors contribute to increased amyloid precursor protein processing and reduce amyloid beta (Aβ) clearance.^11^

Given that AD and VaD exhibit overlapping pathological changes and share similar clinical features, genetic studies may provide the underlying biological links between them. Previous studies have demonstrated that VaD‐associated genes were implicated in AD risk. For example, the *NOTCH3* gene was associated with AD using the c‐alpha test in the United Kingdom and North America.^12^ *NOTCH3* rs149307620, a missense variant, was enriched in AD patients compared to controls in individuals of European ancestry.^13^ However, *NOTCH3* was not associated with AD risk in the Chinese population.^14^, ^15^

To illustrate the role of VaD‐associated genes in the pathogenesis of AD, we comprehensively investigated the associations between these genes and AD risk in a large Chinese cohort via a targeted sequencing panel.

## 2 METHODS

### 2.1 Participants

Our study recruited 1192 AD patients from Xiangya Hospital and 2412 cognitively normal controls from a community in Changsha. AD patients were diagnosed as probable AD by two expert neurologists according to the National Institute on Aging‐Alzheimer's Association criteria for probable AD.^16^ Participants with causative mutations for AD, VaD, and FTD (including *C9orf72*) had been excluded by Sanger sequencing or repeat‐prime PCR (RP‐PCR) analysis. This study was approved by the Ethics Committee of Xiangya Hospital, Central South University, China. Written informed consent was obtained from each participant or guardian.

### 2.2 Genomic DNA isolation

Using phenol–chloroform extraction and ethanol precipitation,^17^ genomic DNA was extracted from the peripheral blood leukocytes of each individual. The quality and quantity of DNA were assessed with a NanoDrop spectrophotometer (Thermo Scientific). All DNA samples were diluted to 50–100 ng/μl.

### 2.3 Gene selection

A detailed literature search in PubMed was manually conducted to select genes associated with VaD. The candidate genes were selected with more than one of the following features: (1) involved in the pathogenesis of VaD; (2) relationship with AD remains controversial; (3) plays role in AD development, such as Aβ metabolism. Eight VaD‐associated genes, *NOTCH3*, *HTRA1*, *TREX1*, *GLA*, *COL4A1*, *CST3*, *GSN*, and *ITM2B*, were finally seclected.^18^, ^19^, ^20^, ^21^, ^22^

### 2.4 Targeted gene sequencing

The targeted sequencing panel comprised eight VaD‐associated genes, namely *NOTCH3*, *HTRA1*, *TREX1*, *GLA*, *COL4A1*, *CST3*, *GSN*, and *ITM2B*. Using Biorupter Pico, the genomic DNA was broken into 150–200‐bp length fragments, followed by end‐repairing, A‐tailing, adaptor ligation, and an 11‐cycle pre‐capture PCR amplification. The fragmented DNA was captured by the targeted panel and sequenced on Illumina NovaSeq 6000 platform. The low‐quality reads fastq data were discarded by FastQC (http://www.bioinformatics.babraham.ac.uk/projects/fastqc/). The paired‐end sequence reads were aligned to the human reference genome (UCSC hg19/GRCH37) using the BWA software (version 0.7.15, http://bio‐bwa.sourceforge.net).^23^ Duplicate sequence reads were removed by Picard (version 2.18.7, http://broadinstitute.github.io/picard/). The quality‐score recalibration, local realignments, and variant calling were performed by the Genome Analysis Toolkit (version 3.2, https://software.broadinstitute.org/gatk/).^24^ Variants were annotated using ANNOVAR (https://hpc.nih.gov/apps/ANNOVAR.html).^25^ Based on minor allele frequencies (MAF), variants were categorized as common or rare variants (common variants: MAF ≥0.01; rare variants: MAF <0.01). Furthermore, ReVe was used to predict the pathogenicity of missense variants.^26^ In our study, the damaging variants were defined as loss‐of‐function (LoF) variants or missense variants with ReVe >0.7. LoF variants included the variants resulting in stop, frameshift, or splice‐site disruption. The variants were named based on the guidelines of the Human Genome Variation Society.^27^

### 2.5 Statistical analysis

With the use of PLINK 1.9,^28^ we filtered out the following variants with genotyping rate <95%, Hardy–Weinberg equilibrium *p*‐value <1 × 10^−6^ in controls, genotype quality (GQ) ≤20, allelic balance departing from 25%/75% ratio of referent and alternate allele reads in the heterozygote, and allelic balance departing from 95% ratio of the homozygote. We performed the common variant‐based association analysis between 1192 AD patients and 2412 cognitively normal controls using PLINK 1.9. Age, gender, and *APOE* ε4 status (*APOE* ε4+, *APOE* ε4−) were adjusted for each common variant. Furthermore, we also performed age at onset (AAO) and Mini‐Mental State Examination (MMSE) association studies in AD patients using the linear regression models in PLINK 1.9.

Additionally, using the Sequence Kernel Association Test‐Optimal (SKAT‐O test),^29^ gene‐based association tests were conducted by combining rare variants between AD patients and cognitively normal controls. Rare variants were further categorized as followings: rare damaging variants (MAF <0.01, LoF or ReVe >0.7), rare damaging missense variants (MAF <0.01, ReVe >0.7), rare LoF variants (MAF <0.01, LoF), and rare missense variants (MAF <0.01, missense). Age, gender, and *APOE* ε4 status were also adjusted by SKAT‐O. Besides, the rare variants association studies were also conducted using PLINK 1.9. A cutoff *p*‐value * *n* < 0.05 was considered statistically significant based on Bonferroni correction (*n* is defined by the number of common variants or genes). Variants or genes not surviving the Bonferroni correction, but with uncorrected *p*‐values less than 0.05, were considered “suggestive.”

### 2.6 VaD genes in the Chinese and European populations

To further investigate the role of VaD genes in the Chinese and European populations, we searched them in AD patients from the webserver AlzData,^30^, ^31^ a freely accessible database in the Chinese population (http://www.alzdata.org/). Meanwhile, the suggestive common variants between AD and controls were also searched in a recent large meta‐genome‐wide association study (GWAS) in the European population.^32^

## 3 RESULTS

### 3.1 Demographic and clinical information

Our study enrolled 1192 AD patients and 2412 cognitively normal controls. The average onset age of AD patients was 63.93 years old, and the average age of controls was 64.76 years old. There was no significant age difference between AD patients and controls (*p* = 0.06). The MMSE scores of AD patients were statistically higher than those of controls (*p* = 4.84×10^−6^). All participants were of southern Han Chinese ancestry (Table 1).

**TABLE 1:** Demographic and clinical information of AD patients and controls

	AD	Control	*p* Value
Number	1192	2412	—
Age (years), mean ± SD	63.93 ± 11.18	64.76±7.77	0.06^a^
Gender (M/F)	475/717	1157/1255	4.84 × 10^−6^ ^b^
MMSE, mean ± SD	12.51 ± 6.77	26.80 ± 2.62	1.20 × 10^−12^ ^a^
MoCA, mean ± SD	8.46 ± 5.13	—	—
CDR, mean ± SD	1.29 ± 0.70	—	—
ADL, mean ± SD	34.41 ± 12.69	—	—
NPI, mean ± SD	18.05 ± 16.13	—	—

Abbreviations: ADL, activities of daily living; CDR, Clinical Dementia Rating; MMSE, Mini‐Mental State Examination; MoCA, Montreal Cognitive Assessment; NPI, Neuropsychiatric Inventory; SD, standard deviation.

^a^ *p*‐Value was calculated by Mann–Whitney *U* test.

^b^ *p*‐Value was calculated by Chi‐squared test.

### 3.2 Common variant association analysis

**FIGURE 1:**
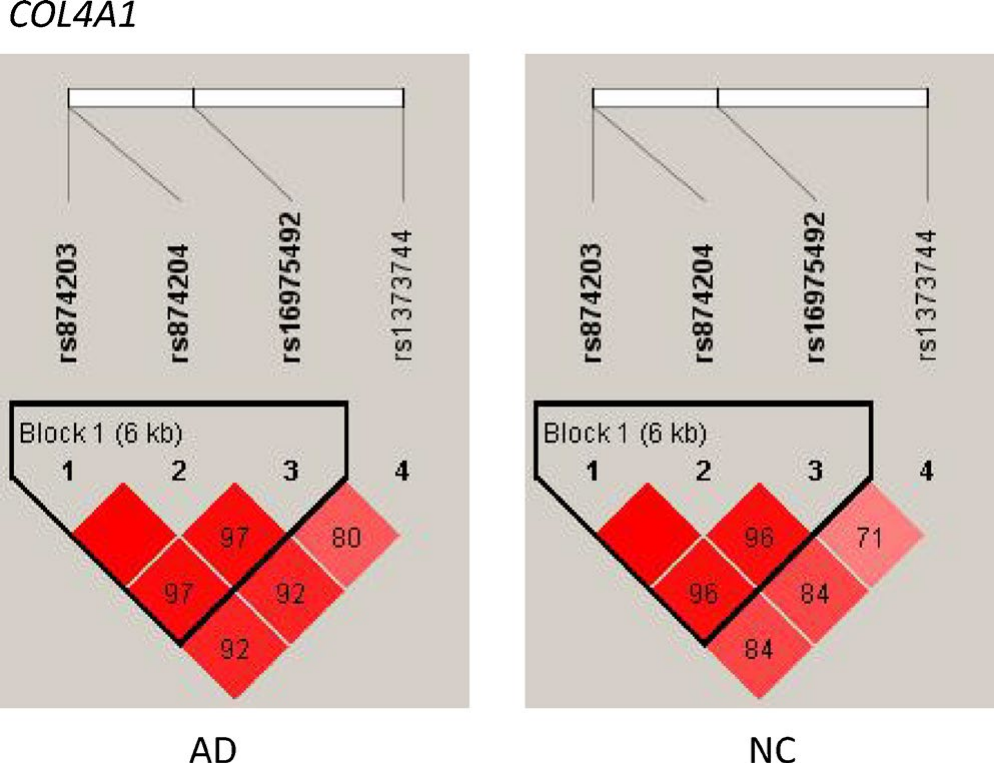
Linkage disequilibrium (LD) patterns of *COL4A1* nominal significant common variants between AD and controls. The value in each square is equal to *r* ^2^ × 100

Forty common variants remained after quality control, including 22 *COL4A1* variants, 11 *NOTCH3* variants, five *GSN* variants, one *HTRA1* variant, and one *ITM2B* variant. These variants were located within exons (62.5%, 25/40), introns (35.0%, 14/40), and 3′‐untranslated regions (3′‐UTRs; 2.5%, 1/40). In the single common variant association test, four *COL4A1* variants were nominally associated with AD risk after the adjustment of age, gender, and *APOE* ε4 status, including rs874203 (*p* = 1.80 × 10^−2^), rs874204 (*p* = 1.84 × 10^−2^), rs16975492 (*p* = 2.34 × 10^−2^), and rs1373744 (*p* = 3.05 × 10^−2^) (Table 2). Nevertheless, after the Bonferroni correction, all these signals were diminished and no longer significant (*p* > 0.00125). The LD patterns of the *COL4A1* variants (rs874203‐rs874204‐rs16975492‐rs1373744) were similar between AD patients and controls (Figure 1). No nominally significant associations were found between the four *COL4A1* variants and AD in a large meta‐GWAS study in the European population (*p* > 0.05).^32^

**TABLE 2:** The nominal significant common variants between AD patients and controls

Gene	Position	Rs ID	Region	Variant	Effect allele	MAF		OR (95% CI)	Adjusted *p*
						Case	Control		
*COL4A1*	13:110827574	rs874203	Exonic	c.3189A > T:p.R1063R	A	0.320	0.293	1.144 (1.023–1.279)	1.80 × 10^−2^
*COL4A1*	13:110827580	rs874204	Exonic	c.3183G > A:p.G1061G	T	0.320	0.293	1.144 (1.023–1.279)	1.84 × 10^−2^
*COL4A1*	13:110833702	rs16975492	Exonic	c.2130G > A:p.P710P	T	0.315	0.289	1.138 (1.018–1.272)	2.34 × 10^−2^
*COL4A1*	13:110843985	rs1373744	Exonic	c.1548A > G:p.Q516Q	T	0.054	0.043	1.299 (1.025–1.646)	3.05 × 10^−2^

Note

Effect allele represents the minor allele.

Abbreviations: adjusted *p*, adjusted by age, gender, and *APOE* ε4 status; CI, confidence interval; MAF, minor allele frequency; OR, odds ratio.

### 3.3 Rare variant aggregation testing

Gene‐based aggregation testing was performed by combining the rare variants within genes. In the rare missense variants group, although the association was nonsignificant after the Bonferroni correction, we observed a suggestive association of *HTRA1* with AD. Specifically, 0.50% of the AD cases and only 0.16% of the controls carried *HTRA1* missense variants (*p* = 4.64 × 10^–2^) (Table 3). In the remaining three groups, including rare damaging variants, rare damaging missense variants, and rare LoF variants, none of the VaD‐associated genes were correlated with AD risk (*p* > 0.05). Additionally, we conducted single rare variant association studies in our cohort. After quality control, 944 rare variants were included in the analysis. None of them reached statistical significance after the Bonferroni correction. Among them, five rare variants showed nominal associations with AD risk, namely *NOTCH3* rs201436750 (*p* = 1.80 × 10^−2^), *COL4A1* rs747972545 (*p* = 2.03 × 10^−2^), *COL4A1* rs201481886 (*p* = 2.41 × 10^−2^), *CST3* rs765692764 (*p* = 2.87 × 10^−2^), and *CST3* rs140837441 (*p* = 4.36 × 10^−2^) (Table 4).

**TABLE 3:** The nominal significant gene between AD patients and controls in the SKAT‐O test

Classification	Gene	Location	Variant	AD (*n*)	Control (*n*)
Rare missense variants (MAF < 0.01)	*HTRA1*	10:124221572	c.404C > A:p.A135D	1	0
		10:124221610	c.442A > C:p.I148L	0	1
		10:124221614	c.446T > C:p.V149A	1	0
		10:124248453	c.508A > C:p.N170H	1	0
		10:124248467	c.522C > G:p.D174E	0	1
		10:124248514	c.569G > A:p.R190H	0	1
		10:124266349	c.920T > C:p.L307P	1	0
		10:124266358	c.929G > A:p.R310H	1	0
		10:124269651	c.1160T > C:p.M387T	1	0
		10:124269662	c.1171A > G:p.T391A	1	1
		10:124269665	c.1174T > C:p.S392P	2	0
		10:124271508	c.1201C > T:p.R401W	2	3
		10:124271513	c.1206C > G:p.H402Q	0	1
		10:124273783	c.1351G > A:p.V451I	1	0
Allele count/total number of alleles (*n*/*n*)				12/2384	8/4824
Frequency (%)				0.50	0.16
Adjusted *p* (SKAT‐O)				4.64×10^−2^	

Abbreviations: adjusted *p*, adjusted by age, gender, and *APOE* ε4 status; SKAT‐O, Sequence Kernel Association Test‐Optimal.

**TABLE 4:** The nominal significant rare variants between AD patients and controls

Gene	Position	Rs ID	Region	Variant	Effect allele	MAF		OR (95% CI)	Adjusted *p*
						Case	Control		
*NOTCH3*	19:15292599	rs201436750	Exonic	c.2580C > T:p.N860N	A	0.003	0.001	5.465 (1.338–22.320)	1.80 × 10^−2^
*COL4A1*	13:110843966	rs747972545	UTR3	c.*7C > T	A	0.005	0.003	2.516 (1.154–5.485)	2.03 × 10^−2^
*COL4A1*	13:110857762	rs201481886	Intronic	—	A	0.002	0.006	0.293 (0.101–0.851)	2.41 × 10^−2^
*CST3*	20:23616002	rs765692764	Exonic	c.246C > T:p.I82I	A	0.003	0.001	4.055 (1.156–14.220)	2.87 × 10^−2^
*CST3*	20:23609225	rs140837441	UTR3	c.*880G > C	G	0.003	0.001	4.330 (1.043–17.970)	4.36 × 10^−2^

Note

Effect allele represents the minor allele.

Abbreviations: adjusted *p*, adjusted by age, gender, and *APOE* ε4 status; CI, confidence interval; OR, odds ratio; UTR, untranslated region.

### 3.4 AAO and MMSE association studies

We performed AAO and MMSE association studies to elucidate the relationships between VaD‐associated genes and AD endophenotypes. In the AAO association study, although no variants reached statistical significance after the Bonferroni correction, five variants were nominally associated with AD, including *GSN* rs9102 (*p* = 1.47 × 10^−2^), *ITM2B* rs11556899 (*p* = 2.65 × 10^−2^), *COL4A1* rs9588116 (*p* = 4.01 × 10^−2^), *COL4A1* rs645114 (*p* = 4.20 × 10^−2^), and *COL4A1* rs9521650 (*p* = 4.53 × 10^−2^). MMSE association study revealed that three variants showed suggestive associations with AD, namely *ITM2B* rs11556899 (*p* = 2.40 × 10^−2^), *GSN* rs12343736 (*p* = 3.32 × 10^−2^), and *GSN* rs2230287 (*p* = 4.12 × 10^−2^) (Table 5).

**TABLE 5:** The nominal significant variants in AAO and MMSE association studies

Classification	Gene	Position	Rs ID	Region	Variant	Effect allele	BETA (95% CI)	Adjusted *p*
AAO association study	*GSN*	9:124094800	rs9102	Exonic	c.2166T > C:p.F722F	C	1.665 (0.329–3.001)	1.47 × 10^−2^
	*ITM2B*	13:48807577	rs11556899	Exonic	c.81C > T:p.L27L	T	2.230 (0.263–4.196)	2.65 × 10^−2^
	*COL4A1*	13:110859069	rs9588116	Intronic	—	C	−1.093 (−2.135‐−0.051)	4.01 × 10^−2^
	*COL4A1*	13:110861785	rs645114	Intronic	—	C	1.083 (0.040–2.127)	4.20 × 10^−2^
	*COL4A1*	13:110866265	rs9521650	Intronic	—	A	1.089 (0.024–2.154)	4.53 × 10^−2^
MMSE association study	*ITM2B*	13:48807577	rs11556899	Exonic	c.81C > T:p.L27L	T	1.565 (0.209–2.921)	2.40 × 10^−2^
	*GSN*	9:124048461	rs12343736	Exonic	c.40T > C:p.W14R	C	−1.068 (−2.050‐−0.087)	3.32 × 10^−2^
	*GSN*	9:124065224	rs2230287	Exonic	c.283G > A:p.A95T	A	−1.027 (−2.010‐−0.043)	4.12 × 10^−2^

Note

Effect allele represents the minor allele.

Abbreviations: AAO, age at onset; adjusted *p*, adjusted by age, gender, and *APOE* ε4 status; BETA, log (odds ratio); CI, confidence interval; MMSE, Mini‐Mental State Examination.

### 3.5 VaD genes in the AlzData database

The association results for rare coding and damaging variants were available in the AlzData database from the whole‐exome sequencing data of Chinese AD patients. Eight VaD‐associated genes were searched in AlzData. In total, we identified 13 variants in *NOTCH3*, one variant in *HTRA1*, three variants in *TREX1*, three variants in *GSN*, one variant in *ITM2B* in the AlzData. None of these variants exhibited associations with AD (*p* > 0.05).

## 4 DISCUSSION

In our study, to explore the role of VaD‐associated genes in AD, eight VaD‐associated genes were screened in a large cohort of AD patients in the Chinese population. We found that the common variants in *COL4A1* were nominally associated with AD. Gene‐based aggregation testing revealed a suggestive association of *HTRA1* with AD. Five rare variants in *NOTCH3*, *COL4A1*, and *CST3* showed nominal associations with AD risk. AAO and MMSE association studies demonstrated that variants in *GSN*, *ITM2B*, and *COL4A1* reached suggestive significance.

VaD refers to a variety of cerebrovascular diseases resulting in cognitive impairment.^33^ Multiple genes were associated with VaD, such as *NOTCH3*,^34^ *HTRA1*,^35^ *GLA*,^36^ and *COL4A1*.^37^ The widely recognized pathological changes include hemorrhages, infarcts, white matter injury, and ischemic brain injury, which were not specific to VaD but also seen in AD.^38^ Accumulating evidence demonstrated that alterations in small or large cerebral vessels were implicated in the development of AD.^39^, ^40^ AD polygenic risk scores were associated with VaD pathological changes, including lobar cerebral microbleeds, white matter lesion load, and artery calcification.^41^ These studies suggested that AD and VaD overlap pathologically and genetically.

Our study found that four *COL4A1* common variants were nominally associated with AD risk. *COL4A1*, located on chromosome 13q34, encodes the α1 chain of type IV collagen. In 2005, *COL4A1* mutations were identified to segregate with human familial porencephaly.^42^ Later, it was recognized that these mutations cause a spectrum of cerebrovascular diseases, ranging from small‐vessel disease to intraparenchymal hemorrhage.^43^, ^44^ A 3′UTR mutation of *COL4A1* caused hereditary multi‐infarct dementia in a Swedish family.^45^ Type IV collagen is a major component of the vascular basement membrane, and *COL4A1* mutations may lead to cortical malformations via vascular insults.^46^ It has been speculated that *COL4A1* mutations perhaps provoke inflammatory reactions and trigger damage to blood vessels, ultimately leading to Aβ deposition.^47^ In a Chinese cohort, the *COL4A1* variant rs3742207 exhibited a marginal association with AD.^15^ Our study showed that *COL4A1* rs874203, rs874204, rs16975492, and rs1373744 were nominally associated with AD risk. For the first time, these variants were identified to be potential contributors to the development of AD in the Chinese population. In the European population, these four *COL4A1* variants showed no associations with AD, which may indicate that they may be Chinese‐specific. However, this result should be interpreted with caution and requires validation in other large Chinese cohorts.

Gene‐based aggregation testing revealed that *HTRA1* exhibited a suggestive association with AD. *HTRA1* is located on chromosome 10q (10q25.3‐q26.2). To date, at least 22 mutations in *HTRA1* have been identified to cause cerebral autosomal recessive arteriopathy with subcortical infarcts and leukoencephalopathy (CARASIL) in an autosomal recessive form.^19^ Most of these mutations potentially lead to increased IGF‐β signaling activity and a reduced level of protease activity, resulting in the degeneration of smooth muscle cells.^48^ Moreover, a significant association was observed between *HTRA1* rs2293871 and cerebral small vessel disease in the elderly.^49^ In 5xFAD mouse analysis and human brain mass spectrometry, *HTRA1* was correlated with Aβ levels.^50^ Specifically, HTRA1 is involved in Aβ metabolism by degrading fragments of amyloid precursor protein.^51^ In the Finland cohort, no significant associations of the *HTRA1* SNPs with AD were observed.^52^ In our study, aggregated rare missense variants of *HTRA1* were nominally associated with AD. Although further studies are warranted to replicate the role of *HTRA1* in AD, our finding indicated that *HTRA1* may exert an effect in the pathogenesis of AD.

The single rare variant association study revealed that five variants were suggestively associated with AD, including variants in *NOTCH3*, *COL4A1*, and *CST3*. Mutations in *NOTCH3* can lead to cerebral autosomal dominant arteriopathy with subcortical infarcts and leukoencephalopathy (CADASIL), the most frequent hereditary cerebral small vessel disease characterized by dementia and stroke.^53^ In a South East Asian cohort, four rare missense variants in *NOTCH3* were marginally associated with AD susceptibility.^15^ We identified that a rare variant in *NOTCH3* achieved suggestive evidence of association with AD, suggesting that *NOTCH3* may confer genetic susceptibility to AD in the Chinese population. Mutations in *CST3* can cause amyloidosis characterized by deposition of abnormal protein fibrils.^54^, ^55^ A meta‐analysis showed that the G73A variant of *CST3* was associated with AD risk in Caucasian populations but not in Asians.^55^ Our study revealed that two rare variants in *CST3* were suggestively associated with AD, indicating that *CST3* may also be a risk gene for AD in the Chinese population.

Substantial evidence indicates that genetic risk factors are involved in AD endophenotypes.^56^, ^57^ To investigate the role of VaD‐associated genes in AD endophenotypes, we performed AAO and MMSE association studies. We found that variants in *GSN*, *ITM2B*, and *COL4A1* were nominally associated with AAO and that variants in *GSN* and *ITM2B* exhibited suggestive associations with MMSE scores. Mutations of *GSN* and *ITM2B* have been identified as the causes of hereditary amyloidosis.^58^ The *GSN* gene encodes gelsolin, which can attenuate the fibrillization of Aβ.^59^ Compared to controls, the plasma GSN levels were significantly declined and positively correlated with MMSE scores in AD patients.^60^ Interestingly, our previous study demonstrated that mutations in *GSN* may contribute to the pathogenesis of AD.^22^ This present study revealed that variants in *GSN* were suggestively correlated not only with AAO but also with MMSE scores, further implicating the *GSN* gene in AD development. *ITM2B* gene encodes integral membrane protein 2B, which can interact with Aβ‐precursor protein and inhibit its processing.^61^ Mutations in *ITM2B* can also lead to rare familial dementias via presynaptic and postsynaptic dysfunction.^62^ Our analysis firstly identified that *ITM2B* variants were marginally associated with AAO and MMSE scores in AD patients, indicating that the *ITM2B* gene may play a role in the pathogenesis of AD.

The nominally significant variants or genes we found have not been reported previously in the AlzData or other Chinese GWASs.^63^, ^64^ Several reasons may contribute to this. First, previous GWASs in the Chinese population focused on variants or genes reaching genome‐wide significance. However, our study only identified suggestive variants or genes that previous studies may not have reported. Second, the Chinese population can be divided into seven population clusters based on principal component analysis.^65^ The sample in our study is mainly from South China, and the diversity of sample sources in China may have resulted in other studies giving different results. Third, differences in sequencing methods between this and previous studies may lead to different findings. Last, although our sample size is large, it is still limited, which may lead to false‐positive or false‐negative results.

In summary, we investigated the role of VaD‐associated genes in AD by comparing AD patients and controls in a large Chinese cohort. The common variant association test demonstrated that *COL4A1* rs874203, rs874204, rs16975492, and rs1373744 were nominally associated with AD. Rare variants in *HTRA1*, *NOTCH3*, *COL4A1*, and *CST3* may also contribute to the etiology of AD. AAO and MMSE association studies implicated variants in *GSN*, *ITM2B*, and *COL4A1* in AD endophenotypes.

## CONFLICT OF INTEREST

The authors declare that there is no conflict of interest associated with the contents of this article.

## Data Availability

The data that support the findings of this study are available from the corresponding author upon reasonable request.
